# Human IgG Subclasses against Enterovirus Type 71: Neutralization *versus* Antibody Dependent Enhancement of Infection

**DOI:** 10.1371/journal.pone.0064024

**Published:** 2013-05-20

**Authors:** Rui-Yuan Cao, Da-Yong Dong, Rui-Ju Liu, Jian-Feng Han, Guang-Chuan Wang, Hui Zhao, Xiao-Feng Li, Yong-Qiang Deng, Shun-Ya Zhu, Xiao-Yu Wang, Fang Lin, Fu-Jun Zhang, Wei Chen, E-De Qin, Cheng-Feng Qin

**Affiliations:** 1 Department of Virology, State Key Laboratory of Pathogen and Biosecurity, Beijing Institute of Microbiology and Epidemiology, Beijing, China; 2 Liaocheng People’s Hospital, Liaocheng, China; 3 The Second Artillery General Hospital, Beijing, China; 4 Rizhao Hospital of Traditional Chinese Medicine, Rizhao, China; University of Rochester, United States of America

## Abstract

The emerging human enterovirus 71 (EV71) represents a growing threat to public health, and no vaccine or specific antiviral is currently available. Human intravenous immunoglobulin (IVIG) is clinical used in treating severe EV71 infections. However, the discovery of antibody dependent enhancement (ADE) of EV71 infection illustrates the complex roles of antibody in controlling EV71 infection. In this study, to identify the distinct role of each IgG subclass on neutralization and enhancement of EV71 infection, different lots of pharmaceutical IVIG preparations manufactured from Chinese donors were used for IgG subclass fractionation by pH gradient elution with the protein A-conjugated affinity column. The neutralization and ADE capacities on EV71 infection of each purified IgG subclass were then assayed, respectively. The neutralizing activity of human IVIG is mainly mediated by IgG1 subclass and to less extent by IgG2 subclass. Interestingly, IgG3 fraction did not have neutralizing activity but enhanced EV71 infection in vitro. These results revealed the different roles of human IgG subclasses on EV71 infection, which is of critical importance for the rational design of immunotherapy and vaccines against severe EV71 diseases.

## Introduction

Human enterovirus type 71 (EV71), a member of the genus *Enterovirus* family *Picornaviridae*, is a typical positive-sense single-stranded RNA virus. A single open reading frame encodes four capsid proteins (VP1–4) and seven nonstructural proteins [1]. EV71 was first isolated from an infant with encephalitis in California, USA in 1969 [2], and then associated with mild diseases and sporadical epidemic globally in the following thirty years. Since last decade, large outbreaks of EV71 infections with severe neurological complications have been reported in Bulgaria, Hungary, Sarawak, Vietnam, Malaysia, Singapore, Korea, Taiwan and Japan [3]. Especially in Asia-Pacific countries, EV71 has emerged as the leading cause of viral encephalitis in infants and young children. Since 2008, millions of EV71 infection cases including thousands of deaths have been reported in mainland China [4], [5]. Considering its global distribution, rapid evolution and neurological tropism [3], EV71 has been recognized as a critical emerging threat to global public health. Unfortunately, there is no vaccine or specific antiviral drugs currently available for EV71 infection.

Various factors, including viral virulence, host susceptibility, humoral and cellular immune response could affect the pathogenesis of EV71 infection. Neutralizing antibodies produced by B cells have been suggested as one of the most important factors in limiting the severity of EV71 infection [6], [7]. Antibody-based therapy has been well demonstrated in animal experiments and clinical practice [7]–[11]. However, a phenomenon termed antibody-dependent enhancement (ADE) was recently confirmed in experimental and clinical settings [12], [13]. Sub-neutralizing concentration of antibodies was evidenced to enhance EV71 infection in Fc receptor-bearing human monocytes and contributed to exacerbation of EV71 infection in mice. Furthermore, the wide existence of cross reactivity between enterovirus antibodies may also become the underlying risk for EV71 ADE infections. Thus, human antibodies appear to play complex roles in controlling EV71 infection. Human IgG antibodies are divided into four subclasses (IgG1–4) based on their distinct Fc segments, and each subclass serves somewhat different functions. Recent studies on dengue and West Nile virus have well demonstrated the different roles of human IgG subclasses in neutralization and ADE activities [14]–[16].

Human intravenous immunoglobulin (IVIG) is a pharmaceutically preparation of human IgG that is pooled from thousands of healthy blood donors, and is evidenced to contain antibodies against various pathogens [17]. Due to the high prevalence of EV71 infection in adults [18]–[20], pharmaceutical IVIG products manufactured from plasma donors in EV71 endemic countries contained high titer EV71 neutralizing antibodies [18], [21]. Herein, we sought to clarify the contribution of different human IgG subclasses to neutralization and enhancement of EV71 infection by using the pharmaceutical IVIG products manufactured from plasma donors in China.

## Materials and Methods

### Cells and Viruses

Human rhabdomyosarcoma (RD) cells were from the American Type Culture Collection (ATCC, no. CCL-136), and cultured with DMEM supplemented with 2% fetal bovine serum (FBS), 100 IU of penicillin, and 100 µg of streptomycin per ml at 37°C in the presence of 5% CO_2_. Human monocytic cells (THP-1) (ATCC no. TIB-202) were used for *in vitro* ADE assay and maintained in RPMI-1640 media at 37°C in 5% CO_2_. EV71 strain AH08/06 (GenBank accession no. HQ611148.1) was isolated from an HFMD patient during an outbreak in 2008 in Anhui, China [22].

### Human IgG Subclasses Preparations

Commercial IVIG products from Chinese donors were kindly provided by Tonrol (Hefei, China) and Ronsen (Chengdu, China) Pharmaceuticals. Human IgG subclasses were fractionated from IVIG products by pH gradient elution with the protein A-conjugated affinity column (Protein A-Sepharose Fast Flow; Amersham Biosciences) and collected by a fast protein liquid chromatography system (AKTA Explorer, Amersham Biosciences) according to the methods modified from previously described [23], [24]. Each IgG subclass fraction was quantified by using the Human IgG Subclass Profile ELISA Kit (Invitrogen), and then concentrated by dialysis to the final concentrations of 2 mg/ml.

### Microneutralization Assay

Microneutralization assays (MN) were performed in human RD cells using EV71 strain AH/08/06 as previously described [22]. Briefly, 50 µL of sample dilutions and 50 µL of virus stock containing 100 TCID_50_ EV71 were mixed and incubated onto the 96-well plates with RD cells at 36°C in a 5% carbondioxide incubator for 6 days. The serial 2-fold IVIG dilutions were tested at an initial dilution of 1∶4, and cell and virus controls were run simultaneously. The neutralizing antibody titer was calculated using the Reed-Muench method [25].

### Antibody-dependent Enhancement (ADE) of Infection Assay

The ADE profile of IgG subclasses was evaluated in human monocytic THP-1 cells as previously described [12], [13]. Briefly, varying concentrations of IgG subclasses and parent IVIG were separately incubated with EV71 for 1 hour at 37°C and then inoculated in the THP-1 cells. After subsequently cultured for 24 h, the viral titer in the supernatant was quantified by using real-time RT-PCR assay. Briefly, the virus RNA in the supernatant was extracted, and one-step real-time RT-PCR was carried out. Absolute quantification of RNA was calculated according to the standard curve, and fold increase of viral titer was calculated accordingly.

## Results

**Figure 1:**
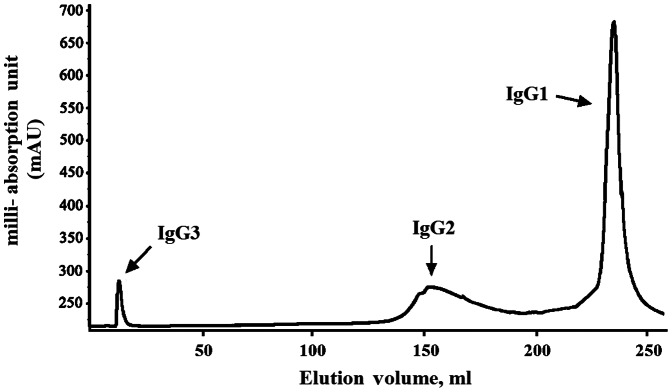
Fractionation of IgG subclasses from human IVIG by FPLC. IgG3 protein was present in the initial ﬂow-through, as indicated. Upon application of a gradually diminishing pH gradient, IgG2 was eluted, followed by IgG1. mAU, milli-Absorption Unit at 280 nm.

In this study, different lots of commercial human IVIG products manufactured from pooled plasma units from healthy Chinese donors were used to fractionate each IgG subclass by pH gradient elution with the protein A-conjugated affinity column. For all preparations, IgG3 was the first fraction that flowed through the column due to the lack of binding ability, and IgG2 was eluted upon application of gradually diminishing pH gradient, followed by IgG1 (Fig. 1). Each fraction of IgG subclass was then quantified by ELISA. The distribution and purity of each IgG subclass were shown in Table 1, and only minor variation were observed among different lots of IVIG preparations.

**Table 1:** Fractionation and purification of human IgG subclasses.

IVIG lots/*IVIG subclass fraction*		ELISA (µg/ml)^a^				Purity (%)			
		IgG1	IgG2	IgG3	IgG4	IgG1	IgG2	IgG3	IgG4
Lot 1		26730.3	15944.6	4114.5	51.5	57.1	34.0	8.8	0.1
	IgG1	2512.8	316.5	46.8	0.0	87.4	11	1.6	0.0
	IgG2	104.0	1170.7	68.2	0.0	7.7	87.2	5.1	0.0
	IgG3	0.0	0.0	467.0	0.0	0.0	0.0	100	0.0
Lot 2		26672.4	18954.6	6315.8	104.0	51.2	36.4	12.1	0.2
Lot 3		32357.7	20795.2	2934.8	2045.0	55.7	35.8	5.0	3.5

a A quantitative IgG subclass-specific ELISA was carried out using the Human IgG Subclass Profile ELISA Kit (Invitrogen).pone.0064024.g004.tif.

**Figure 2:**
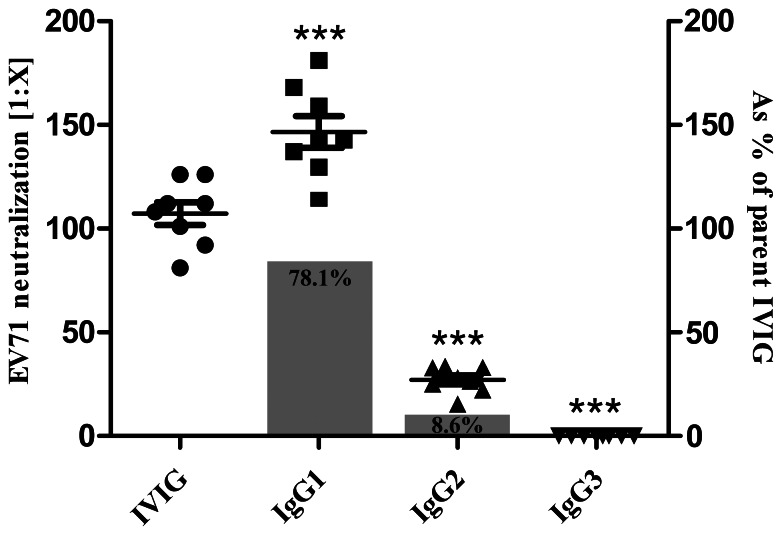
The contribution of each human IgG subclass to EV71 neutralization. Neutralization tests of IgG subclasses were performed using in RD cells for at least 3 times. Error bars represent standard deviation (SD), and statistical analysis was performed by using GraphPad Prism v5.0 software by Student’s t test (unpaired, two-tailed). ***, p<0.005 versus parent IVIG lot.

Each IgG subclass fraction was concentrated by dialysis to the final concentrations of 2 mg/ml before assay. Neutralization assay on human RD cells showed that IgG3 subclass fraction had no neutralization activity against EV71, while IgG1 and IgG2 were both active to neutralize EV71 (Fig. 2). The neutralizing titer of IgG1 subclass fraction is 146.6±21.7, which is significantly higher than that of IgG2 (27.1±6.3), and even the parent IVIG (107.3±15.6). The contribution of each IgG subclass to neutralization capacity of IVIG was then calculated accordingly [15]. As shown in Fig. 2, the majority (89.0%) of EV71 neutralizing capacity of IVIG was due to IgG1 subclass, and IgG2 only played a minor role (7.6%), and IgG3 have no contribution to EV71 neutralization.

**Figure 3:**
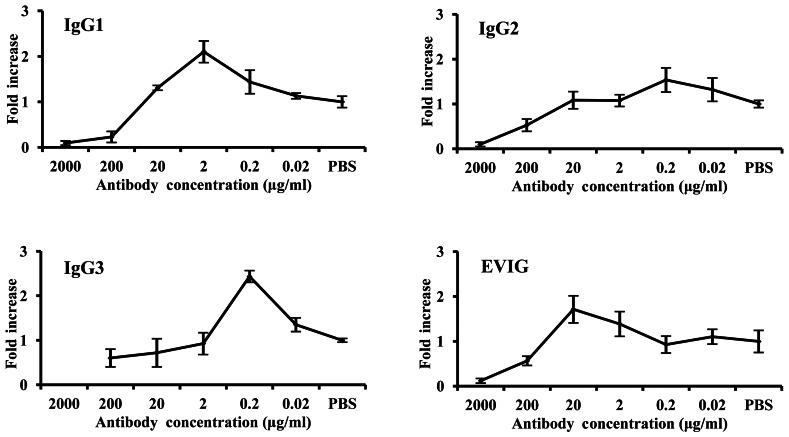
ADE profile of each human IgG subclass against EV71. Ten-fold dilutions of each IgG subclass and parent IVIG was incubated with EV71 strain AH08/06 at an MOI of 1 before incubation with human THP-1 cells, and the ADE potential was represented as fold increase of progeny viral titer compared with the PBS group.

As previously described, IVIG could neutralize EV71 at high concentration (>200 µg/ml), but increased the viral production at a concentration range of 2 to 20 µg/ml (Fig. 2). Significantly, IgG1 and IgG3 subclasses enhanced progeny viral yields at concentrations of 2 and 0.2 µg/ml, respectively. IgG2 showed relatively weak ADE potential, but still, an enhancement of viral production was observed at the concentration of 0.2 µg/ml (Fig. 3). These results revealed the significant different ADE potentials of each human IgG subclasses.

## Discussion

Human IgG subclass response to EV71 infection has not been described yet. The IVIG lots used in this study is manufactured from thousands of Chinese adult plasma donors, thus representing the successful population immune responses against EV71 infection in human [18]. Different lots of IVIG products showed similar IgG subclass profile. Although the exact role of anti-EV71 neutralizing antibody in IVIG is not known yet, commercial IVIG products have been routinely used for severe EV71 infection in Asia [26], [27]. Our results from human IVIG products indicated that IgG1 predominantly determines the neutralizing and protection against EV71. Similarly, immunization with SP70 peptides mainly induced the production of IgG1 in mice [9]. The recently identified EV71 neutralizing monoclonal antibodies E1 and MAB979 belonged to IgG2 and IgG1, respectively [28].

Our results from IVIG products showed that IgG3 presents only enhancing activity without neutralization, whereas IgG2 has weak ADE activity, and IgG1 presents the strongest neutralizing ability. The unique ADE effect of IgG subclasses is in precise correspondence with their affinity with FcγI (CD64) and FcγII (CD32) displayed in the surface of THP-1 cells, indicating the involvement of Fcγs in the ADE infection of EV71 [29], [30]. Although the Fc-receptor mediated enhancement partially explain the phenomenon, the mechanism seem to be much more complex due to: (i) the possibility of involvement of complement activation into the ADE infection [31]; (ii) the polyhedral role of FcγRII in the process of ADE and immune regulation, and this role is made even more complex because of the polymorphism of FcγRII [32]; (iii) the existence of other Fcγ-positive cells like macrophages.

Antibodies mediated neutralization and enhancement depends on the balance between various factors, including viral specie, cell type, antibody concentration, class and subclasss, epitope, etc. PSGL-1 and SCARB2 have recently identified as cellular receptors for EV71 [33], [34]. High resolution crystal structure of EV71 virion and viron-antibody complex will help to understand the mechanism of neutralization and enhancing of EV71 infection. Recently, VP4 was evidenced to contribute to the ADE infection of coxsackieviruses B3, a member of genus *Enterovirus* [35]. Considering the distinct role of antibody and subclass correlation, future study should be warranted to clarify the molecular mechanism of antibody mediated protection and aggravation during EV71 infection.

Our finding is of critical importance for vaccine development and evaluation. Vaccine development for EV71 is particularly active, different vaccine candidates, including inactivated, live attenuated, subunit, subviral particle, and DNA vaccine, have showed immunogenicity and protection in animals. Recently, Chinese FDA has approved large scale clinical trials (http://clinicaltrials.gov/) of inactivated EV71 vaccine. To avoid the potential risk of ADE infection associated with vaccination [36], high level and long duration of neutralizing antibodies must be induced by EV71 vaccine in trials. The IgG subclasses profile in the vaccinated volunteers must be carefully monitored for their neutralizing and ADE activities. Most importantly, the distinct IgG subclass profile against EV71 infection gives critical clue for future vaccine design. Novel vaccine should be designed to induce the production of antibodies with strong neutralizing but weaker ADE activities. Previous studies have demonstrated that IgG3 subclass, which has strong ADE activity but no neutralization activity, was abundantly induced by inactivated vaccine in mice and human [9], [37]. Live attenuated and synthetic peptide based vaccine that induce mainly IgG1 may represent an alternative for future EV71 vaccine development. Besides, vaccine formulation, adjuvant and vaccination methods should be carefully chosen to induce a more beneficial immune response based on the characterized IgG subclass profile.

In conclusion, we successfully demonstrate the human IgG subclasses profile against EV71 infection in commercial human IVIG preparations manufactured in EV71-endemic areas, and clarify the distinct function of each IgG subclass on neutralization and enhancement of EV71 infection. This information is of critically importance for the rational design of antiviral and vaccines against EV71.
